# Discovery of 8-Amino-Substituted 2-Phenyl-2,7-Naphthyridinone Derivatives as New c-Kit/VEGFR-2 Kinase Inhibitors

**DOI:** 10.3390/molecules24244461

**Published:** 2019-12-05

**Authors:** Haiyan Sun, Linsheng Zhuo, Huan Dong, Wei Huang, Nengfang She

**Affiliations:** 1 Nanjing Polytechnic Institute, Nanjing 210048, China; 2 Key Laboratory of Pesticide & Chemical Biology, Ministry of Education, College of Chemistry, Central China Normal University, Wuhan 430079, China; zhuolinsheng199010@126.com (L.Z.); d824002958@163.com (H.D.)

**Keywords:** 2,7-naphthyridone, kinase inhibitor, c-Kit, VEGFR-2

## Abstract

The 2,7-naphthyridone scaffold has been proposed as a novel lead structure of MET inhibitors by our group. To broaden the application of this new scaffold, a series of 8-amino-substituted 2-phenyl-2,7-naphthyridin-1(2*H*)-one derivatives were designed and synthesized. Preliminary biological screening resulted in the discovery of a new lead of c-Kit and VEGFR-2 kinase inhibitors. Compound **9k** exhibited excellent c-Kit inhibitory activity, with an IC_50_ value of 8.5 nM, i.e., it is 38.8-fold more potent than compound **3** (IC_50_ of 329.6 nM). Moreover, the compounds **10l** and **10r** exhibited good VEGFR-2 inhibitory activity, with IC_50_ values of 56.5 and 31.7 nM, respectively, i.e., they are 5.0–8.8-fold more potent than compound **3** (IC_50_ of 279.9 nM). Molecular docking experiments provided further insight into the binding interactions of the new lead compounds with c-Kit and VEGFR-2 kinase. In this study, an 8-amino-substituted 2-phenyl-2,7-naphthyridin-1(2*H*)-one scaffold was identified as the new lead structure of c-Kit and VEGFR-2 kinase inhibitors.

## 1. Introduction

**Figure 1:**
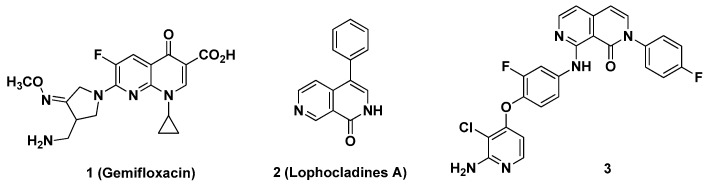
Examples of biologically active compounds containing a naphthyridine scaffold.

*N*-Heterocyclic scaffolds are ubiquitous building blocks that are used for pharmaceuticals and agrochemicals and in materials science. Novel ring structures may exhibit unique biological or physical properties [1]. Naphthyridines, which act as bioisosteres of quinolone, universally exist in nature and form an important class of nitrogen-containing heterocyclic compounds [2]. They possess a conjugated π-system and coplanar stiffness, and are therefore a highly valued building block. Since 1893, a number of naphthyridine derivatives have been synthesized and explored for their biological activities in the search for novel drugs. A broad spectrum of biological activities has been associated with the functional derivatives of naphthyridines [3]. Gemifloxacin (**1**, Figure 1), an antibacterial agent, is the most successful example of a naphthyridine-based drug [4,5]. In recent years, many naphthyridine derivatives have been reported to inhibit protein kinase activity (e.g., PI3K*δ* [6], CK2 [7], Akt1/2 [8], Tpl2 [9,10], and MET [11,12,13]) for the treatment of many types of human diseases, including inflammation and cancer [14,15,16,17,18].

Among the six isomeric sub-classes of naphthyridines, 2,7-naphthyridine derivatives have received increasing attention. A typical pharmaceutically and biologically active derivative is lophocladine A (**2**, Figure 1) [19,20,21,22]. In addition, 2,7-naphthyridine derivatives are widely used as inhibitors of Pdk-1 [23], lumazine synthase [24], PDE-5 [25], TNFα [26], SYK [27], MET [12,13], and other targets. However, the functionalization of 2,7-naphthyridine was found to be especially difficult and only a few methods are available [28], so its application in drug discovery is greatly limited.

**Figure 2:**
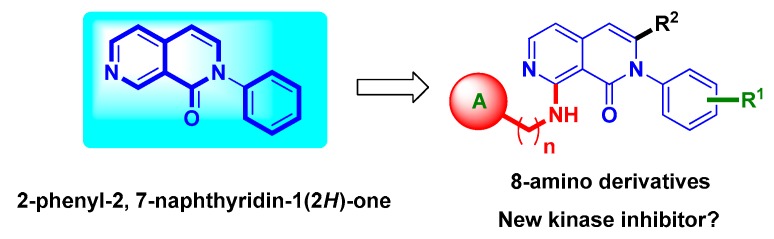
The design of 8-amino-2-phenyl-2,7-naphthyridones.

In our previous work, a novel 2,7-naphthyridone scaffold was designed to conformationally restrain the key pharmacophoric groups of class II MET inhibitors, resulting in the discovery of the potent preclinical candidate compound **3**, which targets MET kinase with a favorable drug-likeness [11]. To further expand the application of the 2,7-naphthyridone scaffold, a series of 8-amino-substituted 2-phenyl-2,7-naphthyridin-1(2*H*)-one derivatives were designed (Figure 2). A small library of 2,7-naphthyridones with structural diversity in its 8-amino groups, substituted 2-phenyl groups, and 3-substituents was constructed to discover new kinase inhibitors.

## 2. Results and Discussion

### 2.1. Chemistry

**Scheme 1:**
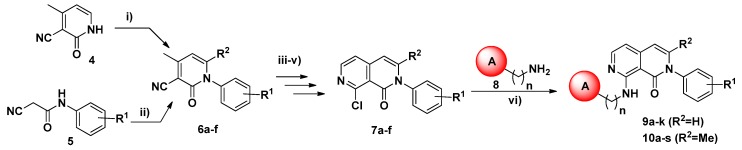
Synthesis of target compounds **9a**–**k** and **10a**–**s**. Reaction conditions and reagents: (i) 1-fluoro-4-iodobenzene, CuI, DMEDA, K_3_PO_4_, dioxane, 100 °C; (ii) 2,4-pentanedione, piperidine, EtOH, 90 °C; (iii) DMF-DMA, DMF, 90 °C; (iv) H_2_SO_4_, 110 °C; (v) POCl_3_, 110 °C; (vi) Pd_2_(dba)_3_, dppp, *t*-BuONa, dioxane, 100 °C.

The synthetic pathway of compounds **9a**–**k** and **10a**–**s** is schematically shown in Scheme 1. According to our reported procedures [11], the key step to constructing the 2-phenyl-2,7-naphthyridone scaffold is the introduction of a 2-phenyl group into the framework. The Ullmann-type coupling of pyridone **4** with 1-fluoro-4-iodobenzene produced 1-phenyl-pyridine-2-one **6a**, while the condensation of 2-cyano-*N*-phenylacetamide **5** with 2,4-pentanedione produced pyridine-2-ones **6b**–**f**. The key building block 8-chloro-2-phenyl-2,7-naphthyridone **7** was successfully produced by a condensation–cyclization–chlorination reaction using substance **6** as the substrate. With intermediate **7** in hand, compounds **9a**–**k** and **10a**–**s** were smoothly synthesized through the direct palladium-catalyzed coupling reactions of substance **7** and the corresponding aromatic amine **8**.

### 2.2. Biological Evaluation

The ability of the synthesized compounds to inhibit MET, c-Kit, and VEGFR-2 activities was evaluated using an enzyme assay with a recombinant kinase domain [29]. Based on the structure activity relationship (SAR) of MET inhibitors [30], we proposed that the removal of the key diaryl ether fragments in compound **3** would result in a loss of MET activity. As shown in Table 1, compounds **9a**–**k** exhibited no obvious MET inhibitory activity at 5000 nM, while our previously reported lead compound **3** exhibited excellent MET inhibitory activity (IC_50_ of 9.9 nM). Interestingly, compound **9g** (*n* = 1, block A-6/4-pyridyl group) exhibited a moderate inhibitory activity against c-Kit (IC_50_ of 832.0 nM) that was only 2.5-fold less potent than that of compound **3** (IC_50_ of 329.6 nM). More importantly, **9k** (*n* = 1, block A-9/4-quinolyl group) exhibited excellent c-Kit inhibitory activity (IC_50_ of 8.5 nM); **9k** is 38.8-fold more potent than compound **3**. Moreover, compounds **9c** (*n* = 0, block A-3/2, 6-dichloro-phenyl group), **9g** (block A-6), and **9k** (block A-9) exhibited moderate VEGFR-2 inhibitory activity (IC_50_ values of 238.5–691.2 nM), which was comparable to compound **3** (IC_50_ of 279.9 nM).

To further study the structure activity relationship (SAR) of the 2-phenyl group and the R^2^ group, compounds **10a**–**s** were screened for their inhibitory activities against MET, c-Kit, and VEGFR-2 (Table 2). Similar to compounds **9a**–**k**, compounds **10a**–**s** showed no obvious MET inhibitory activity. Compound **10d** (2-(4-fluoro)-phenyl group and *n* = 1, block A-9/4-quinolyl group) exhibited weak c-Kit inhibitory activity, while compounds **10l** (2-(4-chloro)-phenyl group) and **10r** (2-(4-trifluoromethyoxy)phenyl group) bearing the same block A-9 (4-quinolyl group) exhibited slightly stronger c-Kit inhibitory activity than compound **3** (IC_50_ of 329.6 nM). Interestingly, most compounds **10** bearing block A-6 (4-pyridyl group) or A-9 (4-quinolyl group) showed different degrees of inhibiting VEGFR-2. For examples, compounds **10d**, **10k**, and **10o** exhibited comparable VEGFR-2 inhibitory activity (IC_50_ values of 208–538 nM) to compound **3** (IC_50_ of 279.9 nM). More importantly, compounds **10l** and **10r** exhibited excellent VEGFR-2 inhibitory activity (IC_50_ values of 31.7–56.5 nM)—i.e., they are 5.0–8.8-fold more potent than compound **3**.

### 2.3. Molecular Modeling

**Figure 3:**
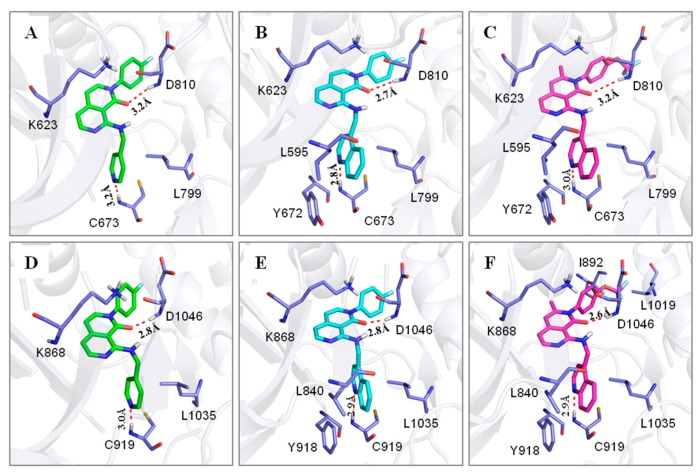
The proposed binding mode of: (**A**) **9g** with c-Kit (green); (**B**) **9k** with c-Kit (cyan); (**C**) **10r** with c-Kit (magenta); (**D**) **9g** with VEGFR-2 (green); (**E**) **9k** with VEGFR-2 (cyan); (**F**) **10r** with VEGFR-2 (magenta). The red dashed line represents a hydrogen bond. For clarity, only key residues are shown.

Molecular docking experiments were further performed to determine the SAR [31,32,33,34,35,36,37]. As shown in Figure 3A–C and Table 3, the entire molecules of **9g**, **9k**, and **10r** were favorably located in the c-Kit binding pocket. The main weak interactions between **9g** and c-Kit included: (1) the H-bond interactions with residues Asp810 and Cys673; (2) the ion–π interaction with Lys623; and (3) the hydrophobic interaction with Leu799. The stronger H-bond interactions with Cys673 and additional hydrophobic interactions with residues Leu595 and Tyr672 of compound **10r** rendered **10r** five-times more potent than compound **9g**. The further enhancement of key H-bond interactions with residues Asp810 and Cys673 resulted in the significantly improved c-Kit inhibitory activity of **9k**.

As shown in Figure 3D–F and Table 3, compounds **9g**, **9k**, and **10r** were entirely located in the VEGFR-2 binding pocket. The main weak interactions between compound **9g** and VEGFR-2 included: (1) the H-bond interactions with residues Asp1046 and Cys919; (2) ion–π interaction with Lys868; (3) the hydrophobic interaction with Leu1035. The stronger H-bond interactions with residues Cys919 and additional hydrophobic interactions with residues Leu840 and Tyr918 rendered compound **9k** more potent than **9g**. The further enhancement of key H-bond interactions with residues Asp1046 and additional hydrophobic interaction with residues Ile892 and Leu1019 resulted in the significantly improved VEGFR-2 inhibitory activity of **10r**.

Taking the molecular docking results into account, we hypothesize that the hydrogen bond acceptor (N containing heterocycle) and hydrophobic effects (fused aromatic ring) of 8-amino substituents are crucial to improve the inhibitory activity for c-Kit and VEGFR-2 kinase.

## 3. Conclusions

In summary, we described the design and synthesis of a series of 8-amino-substituted 2-phenyl-2,7-naphthyridin-1(2*H*)-one derivatives to broaden the application of this new 2,7-naphthyridone scaffold. Preliminary biological screening resulted in the discovery of new lead compounds **9k**, **10l**, and **10r**, which exhibit more potent c-Kit and VEGFR-2 kinase inhibitory activity than the previously reported lead compound, **3**. Molecular docking results provided further insight into the binding interactions of the new lead compounds with c-Kit and VEGFR-2 kinase. We identified 8-amino-substituted 2-phenyl-2,7-naphthyridin-1(2*H*)-one as a new lead scaffold of c-Kit and VEGFR-2 kinase inhibitors.

## 4. Materials and Methods

### 4.1. Biochemical Kinase Assays 

The ability of compounds to inhibit the activity of three kinases (MET, c-Kit, and VEGFR-2) was tested in vitro [30]. Enzyme assays were run in homogeneous time-resolved fluorescence (HTRF) format in 384-well microtiter plates using purified kinases purchased from Invitrogen (Carlsbad, CA, US). The HTRF KinEASE TK kit (contains substrate-biotin, antibody-cryptate, streptavidin-XL665, 5×enzymatic buffer, and detection buffer) was purchased from Cisbio (Codolet, France), and the kinase assays were performed according to the manufacturer’s instructions. After the kinases and the compounds incubated at 25~30 °C for 5 min, the reactions were initiated by the addition of 2 μL of mixed substrate solution (mixed solution of ATP (Sigma, Shanghai, China) and substrate-biotin). The final concentrations of kinases were at EC_80_ and the total reaction volume was 8 μL. Plates were incubated at 30 °C for 30~60 min, then the reactions were quenched by the addition 8 μL mixed detection solution (mixed solution of antibody-cryptate and streptavidin-XL665 in detection buffer). The fluorescence excitation wavelength was 320 nM. The fluorescence at 665 nm (acceptor emission wavelength) and 620 nm (donor emission wavelength) was measured with a PHERAstar FS plate reader (BMG, LABTECH, Ortenberg, Germany) using a time delay of 50 μs. All kinase assays were conducted using ATP concentrations below the enzyme *K*_mapp_ and kinase-specific biotinylated substrate peptides.

The data for dose responses were plotted as percentage of inhibition calculated with the data reduction formula 100 × [1 − (*U*_1_ − *C*_2_)/(*C*_1_ − *C*_2_)] versus concentration of compound, where *U* is the emission ratio of 665 nm and 620 nm of test sample, *C*_1_ is the average value obtained for solvent control (2% DMSO), and *C*_2_ is the average value obtained for no reaction control (no kinase sample). Inhibition curves were generated by plotting percentage of control activity versus log_10_ of the concentration of each kinase. The IC_50_ values were calculated by nonlinear regression with Graphpad Prism 5 (GraphPad Software, San Diego, CA).

### 4.2. Molecular Modeling 

The three-dimensional structures of the small molecules were constructed and primarily optimized by Sybyl 2.0 software. Steepest descent and conjugate gradient methods were used in the optimization process. Autodock Tools was used to assign Gasteiger charges for both the receptors and inhibitors. The optimized probes were docked into the crystal structures of c-Kit (PDB ID: 4U0I) and VEGFR-2 (PDB ID: 3EFL) with Autodock 4.2.5, 1, respectively. The gird size was set to be 70 × 70 × 70 and the grid point spacing was set at default value 0.375 Å. A total of 256 runs were performed by using Lamarkian genetic algorithm (LGA) for conformational search. The best poses were selected for the binding model analysis for all the inhibitors. The figures were prepared with PyMOL 2.2.3 [31,32,33,34,35,36,37].

### 4.3. Chemistry

#### 4.3.1. General Information

Unless otherwise noted, all chemical reagents were commercially available and treated with standard methods. Silica gel column chromatography (CC). Silica gel (200–400 Mesh; Qingdao Makall Group Co., Ltd.; Qingdao; China). Solvents were dried in a routine way and redistilled. All reactions involving air- or moisture-sensitive reagents were performed under a nitrogen or argon atmosphere. ^1^HNMR spectra (400 MHz) and ^13^CNMR (100 MHz) spectra were recorded on a Bruker BioSpin AG (Ultrashield Plus AV 400, Fällanden, Switzerland) spectrometer as deuterochloroform (CDCl_3_) or dimethyl sulfoxide-*d_6_* (DMSO-*d_6_*) solutions using tetramethylsilane (TMS) as an internal standard (*δ* = 0) unless noted otherwise. MS spectra were obtained on an Agilent technologies 6120 quadrupole LC/MS (ESI). All reactions were monitored using thin-layer chromatography (TLC) on silica gel plates. Yields were of purified compounds and were not optimized. 

#### 4.3.2. General Procedure for the Preparation of Intermediates **7a**–**f**

The intermediates **7a**–**f** were prepared according to our previous report [11].

#### 4.3.3. General Procedure for the Preparation of Targets **9a**–**k** and **10a**–**s**

An oven-dried Schlenk tube was charged with **7** (0.4 mmol), Pd_2_(dba)_3_ (0.02 mmol), xantphos (0.04 mmol), *t*-BuONa (0.8 mmol), and amine (0.48 mmol), and then purged with argon three times. Ultra-dry dioxane (15 mL) was added to the Schlenk tube with a syringe at argon atmosphere. The mixture was stirred at 110 °C overnight. After cooling to room temperature, the mixture was concentrated in vacuo and the residue purified by flash chromatography on silica gel using DCM/MeOH (100:1) as eluent to obtain **9a**–**k** and **10a**–**s**.

*2-(4-fluorophenyl)-8-(phenylamino)-2,7-naphthyridin-1(2H)-one* (**9a**): Yellow solid (72% yield). HPLC purity: 98.3%. ^1^H NMR (400 MHz, DMSO-*d*_6_) *δ*: 11.79 (s, 1H), 8.30 (d, *J* = 5.3 Hz, 1H), 7.81 (m, 2H), 7.69 (d, *J* = 7.3 Hz, 1H), 7.61–7.31 (m, 6H), 7.02 (m, 1H), 6.95 (d, *J* = 5.3 Hz, 1H), 6.68 (d, *J* = 7.3 Hz, 1H); ^13^C NMR (100 MHz, DMSO-*d*_6_) *δ*: 162.59, 160.32, 155.89, 150.22, 145.72, 139.81, 137.53, 136.66, 129.47, 128.74, 120.33, 119.81, 109.91, 116.00, 109.91, 105.36; ESI-MS *m*/*z*: 332.3 ([M + H]^+^). 

*8-((2,6-dimethylphenyl)amino)-2-(4-fluorophenyl)-2,7-naphthyridin-1(2H)-one* (**9b**): Yellow solid (82% yield). ^1^H NMR (400 MHz, CDCl_3_) *δ*: 10.57 (s, 1H), 8.14 (d, *J* = 5.6 Hz, 1H), 7.44 (m, 2H), 7.22 (m, 2H); 7.24(d, *J* = 7.2 Hz, 1H), 7.10 (m, 3H), 6.56 (d, *J* = 5.6 Hz, 1H), 6.42 (d, *J* = 7.2 Hz, 1H), 2.23 (s, 6H); ^13^C NMR (100 MHz, DMSO-*d*_6_) *δ*: 162.76, 162.66, 160.22, 157.44, 150.50, 145.73, 137.35, 136.81, 135.23, 129.52, 127.72, 125.91, 116.05, 108.49, 104.96, 104.71, 18.40; ESI-MS *m*/*z*: 360.4 ([M + H]^+^).

*8-((2,6-dichlorophenyl)amino)-2-(4-fluorophenyl)-2,7-naphthyridin-1(2H)-one* (**9c**): Yellow solid (72% yield). HPLC purity: 95.7%. ^1^H NMR (400 MHz, CDCl_3_) *δ*: 10.84 (s, 1H), 8.19 (d, *J =* 5.6 Hz, 1H), 7.43–7.13 (m, 8H), 6.70 (d, *J =* 5.6 Hz, 1H), 6.46 (d, *J =* 7.2 Hz, 1H); ^13^C NMR (100 MHz, DMSO-*d*_6_) *δ*: 162.72, 162.54, 160.28, 156.45, 150.44, 145.62, 137.66, 136.58, 134.56, 133.64, 129.55, 128.50, 116.09, 115.86, 110.07, 105.17, 104.93; ESI-MS *m*/*z*: 401.2 ([M + H]^+^).

*2-(4-fluorophenyl)-8-(pyridin-3-ylamino)-2,7-naphthyridin-1(2H)-one* (**9d**): Yellow solid (85% yield). HPLC purity: 92.1%. ^1^H NMR (400 MHz, DMSO-*d*_6_) *δ*: 11.81 (s, 1H), 8.89 (s, 1H), 8.37 (d, *J* = 8 Hz, 1H), 8.33 (d, *J* = 5.2 Hz, 1H), 8.23 (d, *J* = 3.6 Hz, 1H), 7.71 (d, *J* = 7.2 Hz, 1H), 7.61–7.58 (m, 2H), 7.44–7.35 (m, 3H), 7.03 (d, *J* = 5.2 Hz, 1H), 6.71 (d, *J* = 7.2 Hz, 1H); ^13^C NMR (100 MHz, DMSO-*d*_6_) *δ*: 160.35, 155.77, 150.06, 145.76, 143.02, 141.50, 137.74, 136.58, 129.53, 129.44, 126.57, 123.52, 116.15, 115.92, 110.67, 105.85, 105.17; ESI-MS *m*/*z*: 333.3 ([M + H]^+^).

*2-(4-fluorophenyl)-8-(isoquinolin-7-ylamino)-2,7-naphthyridin-1(2H)-one* (**9e**): Yellow solid (85% yield). HPLC purity: 96.0%. ^1^H NMR (400 MHz, DMSO-*d*6) *δ*: 11.99 (s, 1H), 9.22 (s, 1H), 8.84 (s, 1H), 8.41 (m, 2H), 7.82 (dd, *J*_1_ = 8.8 Hz, *J*_2_ = 8.8 Hz, 2H), 7.56 (d, *J* = 5.2 Hz, 1H), 7.43–7.40 (m, 2H), 7.30 (d, *J* = 7.2 Hz, 1H), 7.28 (d, *J* = 8.8 Hz, 1H), 7.24 (d, *J* = 8.8 Hz, 1H), 6.80(d, *J* = 5.2 Hz, 1H), 6.50 (d, *J* = 7.2 Hz, 1H); ^13^C NMR (100 MHz, DMSO-*d*_6_) *δ*: 162.62, 155.68, 151.44, 150.13, 145.71, 141.72, 138.55, 137.74, 136.60, 131.27, 129.54, 129.19, 127.15, 125.54, 119.98, 116.14, 115.92, 113.83, 110.74, 105.95, 105.24; ESI-MS *m*/*z*: 383.3 ([M + H]^+^).

*8-(benzylamino)-2-(4-fluorophenyl)-2,7-naphthyridin-1(2H)-one* (**9f**): Yellow solid (87% yield). HPLC purity: 96.6%. ^1^H NMR(400 MHz, DMSO-*d*_6_) *δ*: 9.59 (t, *J* = 5.2 Hz, 1H), 8.16 (d, *J* = 5.2 Hz, 1H), 7.59 (d, *J* = 7.2 Hz, 1H), 7.54–7.51 (m, 2H), 7.38–7.25 (m, 7H), 6.70 (d, *J* = 5.2 Hz, 1H), 6.56 (d, *J* = 7.2 Hz, 1H), 4.70 (d, *J* = 5.2 Hz, 2H); ^13^C NMR (100 MHz, DMSO-*d*_6_) *δ*: 162.39, 160.15, 158.40, 151.01, 145.66, 139.62, 137.23, 136.75, 129.33, 128.37, 127.33, 126.78, 115.79, 107.47, 104.85, 104.64, 43.91; ESI-MS *m*/*z*: 346.3 ([M + H]^+^).

*2-(4-fluorophenyl)-8-((pyridin-4-ylmethyl)amino)-2,7-naphthyridin-1(2H)-one* (**9g**): Yellow solid (87% yield). HPLC purity: 99.8%. ^1^H NMR (400 MHz, CDCl_3_) *δ*: 9.71 (t, *J* = 5.6 Hz, 1H), 8.51 (d, *J* = 5.2 Hz, 1H), 8.16 (d, *J* = 5.2 Hz, 1H), 7.39 (d, *J* = 7.2 Hz, 1H), 7.37 (m, 4H), 7.28 (d, *J* = 5.2 Hz, 1H), 7.26–7.18 (m, 2H), 6.56 (d, *J* = 5.2 Hz, 1H), 6.40 (d, *J* = 7.2 Hz, 1H), 4.79 (d, *J* = 5.6 Hz, 2H); ^13^C NMR (100 MHz, DMSO-*d*_6_) *δ*: 162.33, 160.14, 158.33, 150.83, 149.45, 149.14, 145.65, 137.33, 136.72, 129.43, 122.07, 115.98, 107.85, 104.80, 42.77; ESI-MS *m*/*z*: 347.3 ([M + H]^+^).

*2-(4-fluorophenyl)-8-((pyridin-3-ylmethyl)amino)-2,7-naphthyridin-1(2H)-one* (**9h**): Yellow solid (77% yield). HPLC purity: 99.8%. ^1^H NMR (400 MHz, DMSO-*d*6) *δ*: 9.69 (t, *J* = 5.6 Hz, 1H), 8.62 (s, 1H), 8.49 (m, 1H), 8.19 (d, *J* = 5.2 Hz, 1H), 7.79 (m, 1H), 7.63 (d, *J* = 7.2 Hz, 1H), 7.58–7.37 (m, 5H), 6.75 (d, *J* = 5.2 Hz, 1H), 6.60 (d, *J* = 7.2 Hz, 1H), 4.77 (d, *J* = 5.6 Hz, 2H); ^13^C NMR (100 MHz, DMSO-*d*_6_) *δ*: 160.14, 158.29, 150.88, 148.89, 147.79, 145.66, 137.25, 136.71, 135.34, 135.12, 129.40, 128.79, 128.52, 123.46, 115.79, 107.75, 104.83, 41.42; ESI-MS *m*/*z*: 347.3 ([M + H]^+^).

*2-(4-fluorophenyl)-8-((furan-2-ylmethyl)amino)-2,7-naphthyridin-1(2H)-one* (**9i**): Yellow solid (79% yield). HPLC purity: 95.6%. ^1^H NMR (400 MHz, CDCl_3_) *δ*: 9.48 (t, *J* = 5.2 Hz, 1H), 8.22 (d, *J* = 5.2 Hz, 1H), 7.36 (d, *J* = 7.2 Hz, 1H), 7.34–7.32 (m, 2H), 7.21–7.17 (m, 3H), 6.54 (d, *J* = 5.2 Hz, 1H), 6.36 (d, *J* = 7.2 Hz, 1H), 6.27 (m, 2H), 4.74 (d, *J* = 5.2 Hz, 2H); ^13^C NMR (100 MHz, DMSO-*d*_6_) *δ*: 162.59, 162.32, 160.15, 158.05, 152.40, 150.85, 145.62, 142.19, 137.27, 136.69, 129.40, 115.98, 110.43, 107.72, 106.84, 104.83, 104.74, 37.18; ESI-MS *m*/*z*: 336.3 ([M + H]^+^).

*2-(4-fluorophenyl)-8-((naphthalen-1-ylmethyl)amino)-2,7-naphthyridin-1(2H)-one* (**9j**): Yellow solid (81% yield). HPLC purity: 99.5%. ^1^H NMR(400 MHz, DMSO-*d*_6_) *δ*: 9.60 (t, *J* = 5.6 Hz, 1H), 8.19 (d, *J* = 5.2 Hz, 1H), 8.10 (m, 1H), 7.95 (m, 1H), 7.85 (m, 1H), 7.56 (d, *J* = 7.2 Hz, 1H), 7.55–7.43 (m, 6H), 7.31 (m, 2H), 6.71 (d, *J* = 5.2 Hz, 1H), 6.55 (d, *J* = 7.2 Hz, 1H), 5.16 (d, *J* = 5.6 Hz, 2H); ^13^C NMR (100 MHz, DMSO-*d*_6_) *δ*: 145.74, 137.26, 136.67, 136.64, 134.76, 133.38, 131.03, 129.61, 129.37, 129.29, 129.00, 128.55, 127.59, 126.32, 125.81, 125.50, 125.44, 123.39, 115.95, 107.53, 104.88, 104.70, 41.88; ESI-MS *m*/*z*: 396.4 ([M + H]^+^).

*2-(4-fluorophenyl)-8-((quinolin-4-ylmethyl)amino)-2,7-naphthyridin-1(2H)-one* (**9k**): Yellow solid (87% yield). HPLC purity: 99.8%. ^1^H NMR (400 MHz, CDCl_3_) *δ*: 9.72 (t, *J* = 5.6 Hz, 1H), 8.81 (d, *J* = 4.4 Hz, 1H), 8.19 (d, *J* = 5.2 Hz, 1H), 8.13–8.10 (m, 2H), 7.71 (t, *J* = 15.2 Hz, 1H), 7.57 (t, *J* = 15.2 Hz, 1H), 7.44 (d, *J* = 4.4 Hz, 1H), 7.38 (d, *J* = 7.2 Hz, 1H), 7.37–7.35(m, 1H), 7.24–7.17 (m, 3H), 6.58 (d, *J* = 5.2 Hz, 1H), 6.41 (d, *J* = 7.2 Hz, 1H), 5.29 (d, *J* = 5.6 Hz, 2H); ^13^C NMR (100 MHz, DMSO-*d*_6_) *δ*: 162.59, 162.40, 160.15, 158.37, 150.87, 150.31, 147.59, 145.72, 145.12, 137.34, 136.71, 129.61, 129.42, 126.60, 126.16, 123.51, 118.82, 115.98, 115.75, 107.89, 104.91, 40.71; ESI-MS *m*/*z*: 397.4 ([M + H]^+^).

*2-(4-fluorophenyl)-3-methyl-8-(pyridin-3-ylamino)-2,7-naphthyridin-1(2H)-one* (**10a**): Yellow solid (77% yield). HPLC purity: 94.4%. ^1^H NMR (400 MHz, DMSO-*d*_6_) *δ*: 11.72 (s, 1H), 8.87 (s, 1H), 8.36 (m, 1H), 8.25 (d, *J* = 5.6 Hz, 1H), 8.20 (m, 1H), 7.50–7.32 (m, 5H), 6.90 (d, *J* = 5.6 Hz, 1H), 6.64 (s, 1H), 1.98 (s, 3H); ESI-MS *m*/*z*: 347.3 ([M + H]^+^).

*2-(4-fluorophenyl)-3-methyl-8-((pyridin-3-ylmethyl)amino)-2,7-naphthyridin-1(2H)-one* (**10b**): Yellow solid (74% yield). HPLC purity: 96.3%. ^1^H NMR (400 MHz, DMSO-*d*_6_) *δ*: 9.53 (t, *J* = 5.6 Hz, 1H), 8.55 (s, 1H), 8.43 (m, 1H), 8.08 (d, *J* = 5.2 Hz, 1H), 7.72 (m, 1H), 7.40–7.30 (m, 5H), 6.58 (d, *J* = 5.2 Hz, 1H), 6.49 (s, 1H), 4.68 (d, *J* = 5.6 Hz, 2H), 1.92 (s, 3H). ^13^C NMR (100 MHz, DMSO-*d*_6_) *δ*: 162.89, 160.45, 158.09, 150.67, 148.91, 147.96, 145.19, 144.84, 135.39, 135.11, 134.43, 130.85, 130.76, 123.44, 116.33, 107.12, 104.31, 103.39, 21.18; ESI-MS *m*/*z*: 361.4 ([M + H]^+^).

*2-(4-fluorophenyl)-3-methyl-8-((pyridin-4-ylmethyl)amino)-2,7-naphthyridin-1(2H)-one* (**10c**): Yellow solid (77% yield). HPLC purity: 99.8%. ^1^H NMR (400 MHz, DMSO-*d*_6_) *δ*: 9.59 (t, *J* = 5.6 Hz, 1H), 8.46 (d, *J* = 5.0 Hz, 2H), 8.04 (d, *J* = 5.6 Hz, 1H), 7.45–7.35 (m, 4H), 7.28 (d, *J* = 5.0 Hz, 2H), 6.59 (d, *J* = 5.6 Hz, 1H), 6.49 (s, 1H), 4.70 (d, *J* = 5.6 Hz, 2H), 1.94 (s, 3H). ^13^C NMR (100 MHz, DMSO-*d*_6_) *δ*: 160.47, 158.17, 150.63, 149.45, 149.16, 145.20, 144.89, 134.45, 130.88, 130.79, 122.11, 116.34, 107.23, 104.31, 103.44, 42.73, 21.20; ESI-MS *m*/*z*: 361.4 ([M + H]^+^).

*2-(4-fluorophenyl)-3-methyl-8-((quinolin-4-ylmethyl)amino)-2,7-naphthyridin-1(2H)-one* (**10d**): Yellow solid (78% yield). HPLC purity: 95.9%. ^1^H NMR (400 MHz, DMSO-*d*_6_) *δ*: 9.64 (t, *J* = 5.6 Hz, 1H), 8.79 (d, *J* = 4.0 Hz, 1H), 8.21 (m, 1H), 8.06 (d, *J* = 4.0 Hz, 1H), 8.03 (m, 1H), 7.77 (t, *J* = 15.2 Hz, 1H), 7.64 (t, *J* = 15.2 Hz, 1H), 7.44 (d, *J* = 5.2 Hz, 1H), 7.42–7.34 (m, 4H), 6.60 (d, *J* = 5.2 Hz, 1H), 6.50 (s, 1H), 5.21 (d, *J* = 5.6 Hz, 2H), 1.94 (s, 3H). ^13^C NMR (100 MHz, DMSO-*d*_6_) *δ*: 163.60, 162.91, 160.47, 158.14, 150.61, 147.58, 145.27, 144.98, 134.42, 130.86, 129.23, 126.61, 126.17, 124.20, 123.53, 118.90, 118.38, 116.35, 107.27, 104.35, 103.54, 40.64, 21.21; ESI-MS *m*/*z*: 411.4 ([M + H]^+^).

*3-methyl-2-phenyl-8-(pyridin-3-ylamino)-2,7-naphthyridin-1(2H)-one* (**10e**): Yellow solid (80% yield). HPLC purity: 90.1%. ^1^H NMR (400 MHz, DMSO-*d*_6_) *δ*: 11.76 (s, 1H), 8.86 (s, 1H), 8.36 (m, 1H), 8.25 (d, *J* = 5.2 Hz, 1H), 8.19 (m, 1H), 7.64–7.50 (m, 4H), 7.39 (m, 1H), 7.34 (m, 1H), 6.90 (d, *J* = 5.2 Hz, 1H), 6.63 (s, 1H), 1.98 (s, 3H); ^13^C NMR (100 MHz, DMSO-*d*_6_) *δ*: 164.14, 158.29, 150.68, 145.37, 142.82, 141.34, 138.04, 136.64, 132.19, 131.50, 129.56, 128.67, 126.38, 123.48, 110.01, 104.71, 104.47, 21.23; ESI-MS *m*/*z*: 329.3 ([M + H]^+^).

*3-methyl-2-phenyl-8-((pyridin-3-ylmethyl)amino)-2,7-naphthyridin-1(2H)-one* (**10f**): Yellow solid (78% yield). HPLC purity: 97.9%. ^1^H NMR (400 MHz, DMSO-*d*_6_) *δ*: 9.55 (t, *J* = 4.8 Hz, 1H), 8.55 (m, 1H), 8.43 (m, 1H), 8.08 (d, *J* = 4.4 Hz, 1H), 7.72–7.31 (m, 7H), 6.59 (d, *J* = 4.4 Hz, 1H), 6.48 (s, 1H), 4.68 (d, *J* = 4.8 Hz, 2H), 1.92 (s, 3H); ^13^C NMR (100 MHz, DMSO-*d*_6_) *δ*: 163.44, 158.12, 150.60, 148.91, 147.97, 145.17, 144.80, 138.23, 135.38, 135.10, 129.39, 128.55, 123.45, 107.11, 104.24, 103.45, 41.35, 21.18; ESI-MS *m*/*z*: 343.4 ([M + H]^+^).

*3-methyl-2-phenyl-8-((pyridin-4-ylmethyl)amino)-2,7-naphthyridin-1(2H)-one* (**10g**): Yellow solid (67% yield). HPLC purity: 97.7%. ^1^H NMR (400 MHz, DMSO-*d*_6_) *δ*: 9.60 (t, *J* = 5.6 Hz, 1H), 8.46 (d, *J* = 5.0 Hz, 2H), 8.04 (d, *J* = 5.6 Hz, 1H), 7.56–7.46 (m, 3H), 7.34 (m, 2H), 7.28 (d, *J* = 5.0 Hz, 2H), 6.59 (d, *J* = 5.6 Hz, 1H), 6.49 (s, 1H), 4.70 (d, *J* = 5.6 Hz, 2H), 1.93 (s, 3H); ^13^C NMR (100 MHz, DMSO-*d*_6_) *δ*: 163.47, 158.19, 150.55, 149.44, 149.17, 145.18, 144.85, 138.24, 129.40, 128.58, 126.56, 122.15, 107.23, 104.23, 103.51, 42.74, 21.19; ESI-MS *m*/*z*: 343.4 ([M + H]^+^).

*3-methyl-2-phenyl-8-((quinolin-4-ylmethyl)amino)-2,7-naphthyridin-1(2H)-one* (**10h**): Yellow solid (88% yield). ^1^H NMR (400 MHz, DMSO-*d*_6_) *δ*: 9.66 (t, *J* = 5.6 Hz, 1H), 8.79 (d, *J* = 4.0 Hz, 1H), 8.21 (m, 1H), 8.06 (d, *J* = 4.0 Hz, 1H), 8.03 (d, *J* = 5.2 Hz, 1H), 7.77 (t, *J* = 15.2 Hz, 1H), 7.64 (t, *J* = 15.2 Hz, 1H), 7.56–7.33 (m, 6H), 6.60 (d, *J* = 5.2 Hz, 1H), 6.51 (s, 1H), 5.20 (d, *J* = 5.6 Hz, 2H), 1.94 (s, 3H); ESI-MS *m*/*z*: 393.4 ([M + H]^+^).

*2-(4-chlorophenyl)-3-methyl-8-(pyridin-3-ylamino)-2,7-naphthyridin-1(2H)-one* (**10i**): Yellow solid (86% yield). HPLC purity: 91.1%. ^1^H NMR (400 MHz, DMSO-*d*_6_) *δ*: 11.70 (s, 1H), 8.86 (s, 1H), 8.36 (m, 1H), 8.25 (d, *J* = 5.2 Hz, 1H), 8.19 (m, 1H), 7.82–7.46 (m, 4H), 7.39 (m, 1H), 6.90 (d, *J* = 5.2 Hz, 1H), 6.65 (s, 1H), 1.98 (s, 3H); ^13^C NMR (100 MHz, DMSO-*d*_6_) *δ*:163.14, 155.29, 149.89, 145.37, 142.82, 141.34, 138.04, 136.64, 132.19, 131.50, 129.56, 128.67, 126.38, 123.48, 110.01, 104.71, 104.47, 21.23; ESI-MS *m*/*z*: 363.8 ([M + H]^+^).

*2-(4-chlorophenyl)-3-methyl-8-((pyridin-3-ylmethyl)amino)-2,7-naphthyridin-1(2H)-one* (**10j**): Yellow solid (74% yield). HPLC purity: 92.1%. ^1^H NMR (400 MHz, DMSO-*d*_6_) *δ*: 9.50 (t, *J* = 5.6 Hz, 1H), 8.55 (s, 1H), 8.43 (d, *J* = 4.4 Hz, 1H), 8.08 (d, *J* = 5.2 Hz, 1H), 7.71 (d, *J* = 7.6 Hz, 1H), 7.61 (d, *J* = 8.4 Hz, 2H), 7.39 (d, *J* = 8.4 Hz, 2H), 7.32 (dd, *J*_1_ = 4.4 Hz, *J*_2_ = 7.6 Hz, 1H), 6.58 (d, *J* = 5.2 Hz, 1H), 6.49 (s, 1H), 4.68 (d, *J* = 5.6 Hz, 2H), 1.92 (s, 3H); ^13^C NMR (100 MHz, DMSO-*d*_6_) *δ*: 163.40, 158.08, 150.73, 148.88, 147.97, 145.21, 144.59, 137.09, 135.37, 135.14, 133.20, 130.63, 129.43, 123.47, 107.17, 104.42, 103.34, 41.35, 21.13; ESI-MS *m*/*z*: 377.8 ([M + H]^+^).

*2-(4-chlorophenyl)-3-methyl-8-((pyridin-4-ylmethyl)amino)-2,7-naphthyridin-1(2H)-one* (**10k**): Yellow solid (77% yield). HPLC purity: 99.8%. ^1^H NMR (400 MHz, DMSO-*d*_6_) *δ*: 9.59 (t, *J* = 5.6 Hz, 1H), 8.46 (d, *J* = 4.0 Hz, 2H), 8.04 (d, *J* = 5.6 Hz, 1H), 7.61 (d, *J* = 8.4 Hz, 2H), 7.41 (d, *J* = 8.4 Hz, 2H), 7.28 (d, *J* = 5.0 Hz, 2H), 6.59 (d, *J* = 5.6 Hz, 1H), 6.49 (s, 1H), 4.70 (d, *J* = 5.6 Hz, 2H), 1.94 (s, 3H); ^13^C NMR (100 MHz, DMSO-*d*_6_) *δ*: 163.42, 158.15, 150.68, 149.44, 149.15, 145.21, 144.63, 137.11, 133.21, 130.66, 129.43, 122.13, 107.27, 104.41, 103.40, 42.74, 21.15; ESI-MS *m*/*z*: 377.8 ([M + H]^+^).

*2-(4-chlorophenyl)-3-methyl-8-((quinolin-4-ylmethyl)amino)-2,7-naphthyridin-1(2H)-one* (**10l**): Yellow solid (84% yield). HPLC purity: 98.0%. ^1^H NMR (400 MHz, DMSO-*d*_6_) *δ*: 9.62 (t, *J* = 5.6 Hz, 1H), 8.79 (d, *J* = 4.0 Hz, 1H), 8.21 (m, 1H), 8.06 (d, *J* = 5.2 Hz, 1H), 8.03 (m, 1H), 7.77 (t, *J* = 15.2 Hz, 1H), 7.64 (t, *J* = 15.2 Hz, 1H), 7.61 (d, *J* = 8.4 Hz, 2H), 7.40 (d, *J* = 8.4 Hz, 2H), 7.37 (d, *J* = 4.0 Hz, 1H), 6.60 (d, *J* = 5.2 Hz, 1H), 6.51 (s, 1H), 5.21 (d, *J* = 5.6 Hz, 2H), 1.94 (s, 3H); ^13^C NMR (100 MHz, DMSO-*d*_6_) *δ*: 162.91, 158.14, 150.73, 150.31, 147.60, 145.28, 145.15, 144.69, 137.10, 133.22, 130.65, 129.61, 129.44, 129.23, 126.62, 126.17, 123.52, 118.91, 107.30, 104.44, 103.49, 40.63, 21.16; ESI-MS *m*/*z*: 427.9 ([M + H]^+^).

*3-methyl-8-(pyridin-3-ylamino)-2-(4-(trifluoromethoxy)phenyl)-2,7-naphthyridin-1(2H)-one* (**10m**)*:* Yellow solid (79% yield). HPLC purity: 99.1%. ^1^H NMR (400 MHz, DMSO-*d*_6_) *δ*: 11.67 (s, 1H), 8.87 (s, 1H), 8.36 (d, *J* = 8.0 Hz, 1H), 8.25 (d, *J* = 5.6 Hz, 1H), 8.20 (d, *J* = 4.0 Hz, 1H), 7.59 (s, 4H), 7.34 (dd, *J*_1_ = 8.0 Hz, *J*_2_ = 4.0 Hz, 1H), 6.90 (d, *J* = 5.6 Hz, 1H), 6.65 (s, 1H), 1.98 (s, 3H); ^13^C NMR (100 MHz, DMSO-*d*_6_) *δ*: 163.62, 155.54, 149.94, 148.24, 145.29, 142.88, 141.38, 136.99, 136.58, 130.83, 126.45, 123.49, 122.14, 121.31, 118.76, 110.05, 104.91, 104.38, 21.20; ESI-MS *m*/*z*: 413.3 ([M + H]^+^).

*3-methyl-8-((pyridin-3-ylmethyl)amino)-2-(4-(trifluoromethoxy)phenyl)-2,7-naphthyridin-1(2H)-one* (**10n**): Yellow solid (74% yield). ^1^H NMR (400 MHz, DMSO-*d*_6_) *δ*: 9.50 (t, *J* = 5.6 Hz, 1H), 8.55 (s, 1H), 8.43 (d, *J* = 4.0 Hz, 1H), 8.08 (d, *J* = 5.2 Hz, 1H), 7.72 (d, *J* = 7.6 Hz, 1H), 7.59 (s, 4H), 7.32 (dd, *J*_1_ = 4.0 Hz, *J*_2_ = 7.6 Hz, 1H), 6.58 (d, *J* = 5.2 Hz, 1H), 6.50 (s, 1H), 4.68 (d, *J* = 5.6 Hz, 2H), 1.93 (s, 3H); ^13^C NMR (100 MHz, DMSO-*d*_6_) *δ*: 163.43, 158.08, 150.78, 148.92, 148.06, 147.98, 145.22, 144.52, 137.21, 135.36, 130.89, 123.44, 121.97, 121, 30, 107.14, 104.43, 103.33, 41.34, 21.14; ESI-MS *m*/*z*: 427.4 ([M + H]^+^).

*3-methyl-8-((pyridin-4-ylmethyl)amino)-2-(4-(trifluoromethoxy)phenyl)-2,7-naphthyridin-1(2H)-one* (**10o**): Yellow solid (79% yield). HPLC purity: 99.4%. ^1^H NMR (400 MHz, DMSO-*d*_6_) *δ*: 9.56 (t, *J* = 5.6 Hz, 1H), 8.46 (d, *J* = 5.2 Hz, 2H), 8.04 (d, *J* = 5.2 Hz, 1H), 7.54 (s, 4H), 7.28 (d, *J* = 5.2 Hz, 2H), 6.59 (d, *J* = 5.2 Hz, 1H), 6.50 (s, 1H), 4.70 (d, *J* = 5.6 Hz, 2H), 1.95 (s, 3H); ^13^C NMR (100 MHz, DMSO-*d*_6_) *δ*: 163.18, 158.36, 150.71, 149.39, 148.08, 145.50, 144.58, 137.21, 130.97, 123.85, 122.13, 121.30, 118.75, 107.27, 104.44, 103.38, 42.74, 21.16; ESI-MS *m*/*z*: 427.4 ([M + H]^+^).

*8-((furan-2-ylmethyl)amino)-3-methyl-2-(4-(trifluoromethoxy)phenyl)-2,7-naphthyridin-1(2H)-one* (**10p**): Yellow solid (82% yield). HPLC purity: 96.3%. ^1^H NMR (400 MHz, DMSO-*d*_6_) *δ*: 9.32 (t, *J* = 5.6 Hz, 1H), 8.12 (d, *J* = 5.6 Hz, 1H), 7.55–7.49 (m, 5H), 6.60 (d, *J* = 5.6 Hz, 1H), 6.50 (s, 1H), 6.37 (m, 1H), 6.28 (m, 1H), 4.65 (d, *J* = 5.6 Hz, 2H), 1.93 (s, 3H); ^13^C NMR (100 MHz, DMSO-*d*_6_) *δ*: 163.43, 157.84, 152.41, 150.76, 148.07, 145.20, 144.53, 142.19, 137.18, 130.87, 121.98, 110.42, 107.13, 106.85, 104.48, 103.31, 66.33, 37.10, 21.15; ESI-MS *m*/*z*: 416.3 ([M + H]^+^).

*3-methyl-8-((naphthalen-1-ylmethyl)amino)-2-(4-(trifluoromethoxy)phenyl)-2,7-naphthyridin-1(2H)-one* (**10q**): Yellow solid (79% yield). HPLC purity: 97.0%. ^1^H NMR (400 MHz, DMSO-*d*_6_) *δ*: 9.43 (t, *J* = 5.6 Hz, 1H), 8.15 (d, *J* = 5.2 Hz, 1H), 8.10 (d, *J* = 8.4 Hz, 1H), 7.93 (dd, *J*_1_ = 8.4 Hz, *J*_2_ = 8.0 Hz, 1H), 7.84 (d, *J* = 8.0 Hz, 1H), 7.54–7.42 (m, 8H), 6.60 (d, *J* = 5.2 Hz, 1H), 6.50 (s, 1H), 5.13 (d, *J* = 5.6 Hz, 2H), 1.91 (s, 3H);^13^C NMR(100 MHz, DMSO-*d*_6_)*δ*: 158.06, 150.94, 148.01, 145.29, 144.50, 137.14, 134.82, 133.37, 131.03, 130.83, 128.55, 127.59, 126.32, 125.80, 125.50, 123.39, 121.93, 121.26, 118.71, 106.93, 104.52, 103.24, 41.76, 21.14; ESI-MS *m*/*z*: 476.4 ([M + H]^+^).

*3-methyl-8-((quinolin-4-ylmethyl)amino)-2-(4-(trifluoromethoxy)phenyl)-2,7-naphthyridin-1(2H)-one* (**10r**): Yellow solid (80% yield). HPLC purity: 94.0%. ^1^H NMR (400 MHz, DMSO-*d*_6_) *δ*: 9.62 (t, *J* = 5.6 Hz, 1H), 8.81 (d, *J* = 4.0 Hz, 1H), 8.21 (m, 1H), 8.06 (d, *J* = 5.2 Hz, 1H), 8.05 (m, 1H), 7.77 (t, *J* = 15.2 Hz, 1H), 7.64 (t, *J* = 15.2 Hz, 1H), 7.55 (s, 4H), 7.37 (d, *J* = 4.0 Hz, 1H), 6.63 (d, *J* = 5.2 Hz, 1H), 6.54 (s, 1H), 5.22 (d, *J* = 5.6 Hz, 2H), 1.96 (s, 3H); ^13^C NMR (100 MHz, DMSO-*d*_6_) *δ*: 163.12, 158.66, 151.28, 150.80, 148.12, 145.80, 145.64, 145.12, 137.69, 131.40, 130.12, 129.71, 127.10, 126.68, 124.02, 122.46, 119.43, 107.80, 104.98, 103.99, 41.12, 21.66; ESI-MS *m*/*z*: 477.4 ([M + H]^+^).

*2-(2,4-difluorophenyl)-3-methyl-8-((quinolin-4-ylmethyl)amino)-2,7-naphthyridin-1(2H)-one* (**10s**): Yellow solid (88% yield). HPLC purity: 99.1%. ^1^H NMR (400 MHz, DMSO-*d*_6_) *δ*: 9.54 (t, *J* = 5.6 Hz, 1H), 8.80 (d, *J* = 4.4 Hz, 1H), 8.21 (d, *J* = 8.0 Hz, 1H), 8.09 (d, *J* = 5.6 Hz, 1H), 8.05 (d, *J* = 8.0 Hz, 1H), 7.77 (t, *J* = 15.2 Hz, 1H), 7.64 (t, *J* = 15.2 Hz, 1H), 7.61 (s, 1H), 7.55 (t, *J* = 14.8 Hz, 1H), 7.36 (d, *J* = 4.4 Hz, 1H), 7.29 (t, *J* = 14.8 Hz, 1H), 6.63 (d, *J* = 5.6 Hz, 1H), 6.56 (s, 1H), 5.21 (d, *J* = 5.6 Hz, 2H), 1.99 (s, 3H); ^13^C NMR (100 MHz, DMSO-*d*_6_) *δ*: 163.02, 158.10, 151.19, 150.32, 147.59, 145.41, 145.09, 144.56, 132.19, 132.09, 129.60, 129.23, 126.61, 126.16, 123.51, 122.04, 118.88, 122.57, 107.41, 105.31, 105.04, 104.80, 103.05, 40.68, 20.51; ESI-MS *m*/*z*: 429.4 ([M + H]^+^).

## Figures and Tables

**Table 1:**
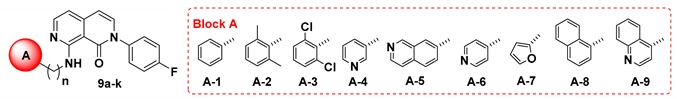
Inhibitory activity of **9a–k** against MET, c-Kit, and VEGFR-2.

No.	Block A	n	Inhibitory Activity, IC_50_, nM ^a^		
			MET	c-Kit	VEGFR-2
**9a**	A-1	0	>5000	>5000	>5000
**9b**	A-2	0	>5000	>5000	>5000
**9c**	A-3	0	>5000	>5000	691.2
**9d**	A-4	0	>5000	>5000	>5000
**9e**	A-5	0	>5000	>5000	>5000
**9f**	A-1	1	>5000	>5000	>5000
**9g**	A-6	1	>5000	832.0	601.3
**9h**	A-4	1	>5000	>5000	>5000
**9i**	A-7	1	>5000	>5000	>5000
**9j**	A-8	1	>5000	>5000	>5000
**9k**	A-9	1	>5000	8.5	238.5
**3**			9.9	329.6	279.9

^a^ In vitro kinase assays were performed with the indicated purified recombinant MET, c-Kit, or VEGFR-2 kinase domains (nM).

**Table 2:**
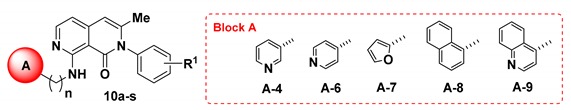
Inhibitory activity of **10a–s** against MET, c-Kit, and VEGFR-2.

No.	Block A	n	R^1^	Inhibitory Activity, IC_50_, nM ^a^		
				c-Met	c-Kit	VEGFR-2
**10a**	A-4	0	4-F	>5000	>5000	>5000
**10b**	A-4	1	4-F	>5000	>5000	>5000
**10c**	A-6	1	4-F	>5000	>5000	>5000
**10d**	A-9	1	4-F	>5000	1609	208
**10e**	A-4	0	H	>5000	>5000	>5000
**10f**	A-4	1	H	>5000	>5000	>5000
**10g**	A-6	1	H	>5000	>5000	>5000
**10h**	A-9	1	H	>5000	>5000	1031
**10i**	A-4	0	4-Cl	>5000	>5000	>5000
**10j**	A-4	1	4-Cl	>5000	>5000	>5000
**10k**	A-6	1	4-Cl	>5000	>5000	263
**10l**	A-9	1	4-Cl	>5000	107	56.5
**10m**	A-4	0	4-OCF_3_	>5000	>5000	>5000
**10n**	A-4	1	4-OCF_3_	>5000	>5000	>5000
**10o**	A-6	1	4-OCF_3_	>5000	>5000	538
**10p**	A-7	1	4-OCF_3_	>5000	>5000	>5000
**10q**	A-8	1	4-OCF_3_	>5000	>5000	>5000
**10r**	A-9	1	4-OCF_3_	>5000	169	31.7
**10s**	A-9	1	2,4-F_2_	>5000	>5000	887
**3**				9.9	329.6	279.9

^a^ In vitro kinase assays were performed with the indicated purified recombinant MET, c-Kit, or VEGFR-2 kinase domains (nM).

**Table 3:** Individual energy components of the free energies of ligand with c-Kit and VEGFR-2.

N.O.	Compound	ΔE_ele_	ΔE_vdw_	ΔG_np_	ΔG_pol_	ΔH	-TΔS	ΔG_cal_	IC_50_ (nM)
c-Kit (4U0I)	**9g**	−18.60	−52.83	−5.49	42.12	−34.81	14.67	−20.13	832
	**9k**	−21.78	−60.09	−5.91	44.37	−43.42	13.19	−30.23	8.5
	**10r**	−18.78	−59.91	−6.52	45.23	−39.98	15.90	−24.08	169
VEGFR-2 (3EFL)	**9g**	−26.32	−53.91	−5.36	40.44	−45.15	15.18	−29.97	601
	**9k**	−26.56	−60.46	−5.86	42.55	−50.34	15.55	−34.79	238.5
	**10r**	−27.82	−64.08	−6.37	43.36	−54.92	16.38	−38.54	31.7

## Abbreviations

MET: mesenchymal−epithelial transition factor; c-Kit, receptor tyrosine kinase for the stem cell factor; VEGFR-2, vascular endothelial growth factor receptor 2; PI3K*δ*, phosphatidylinositol 3-kinase delta; CK2, protein kinase CK2; Akt1/2, protein kinase B1/2; Tpl-2, Tumor Progression Loci-2; Pdk-1, phosphoinositide-dependent protein kinase-1; PDE-5, phosphodiesterase type 5; TNFα, tumor necrosis factor alpha; SYK, spleen tyrosine kinase; DMEDA, *N*,*N*’-Dimethyl-1,2-ethanediamine; DMF-DMA, *N*,*N*-dimethylformamide dimethyl acetal; DMF, *N*,*N*-dimethylformamide; ATP, adenosine triphosphate; DMSO, dimethyl sulfoxide; HNMR, hydrogen nuclear magnetic resonance; CNMR, carbon nuclear magnetic resonance; MS, mass spectrum; LC/MS (ESI), liquid chromatography-mass spectrometry (Electrospray ionization).

## References

[1] Greiner R., Ziegler D.S., Cibu D., Jakowetz A.C., Auras F., Bein T., Knochel P. (2017). Preparation of polyfunctional naphthyridines by cobalt-catalyzed cross-couplings of halogenated naphthyridines with magnesium and zinc organometallics. Org. Lett..

[2] Brown D.J., Ellman J.A., Taylor E.C. (2007). The Naphthyridines.

[3] Litvinov V.P., Roman S.V., Dyachenko V.D. (2000). Naphthyridines. Structure, physicochemical properties and general methods of synthesis. Russ. Chem. Rev..

[4] Paim C.S., Araujo B.V., Volpato N.M., Steppe M., Schapoval E.E.S. (2015). Gemifloxacin mesylate (GFM): Dissolution test based on in vivo data. Drug Dev. Ind. Pharm..

[5] Chen T.C., Hsu Y.L., Tsai Y.C., Chang Y.W., Kuo P.L., Chen Y.H. (2014). Gemifloxacin inhibits migration and invasion and induces mesenchymal–epithelial transition in human breast adenocarcinoma cells. J. Mol. Med..

[6] Norman P. (2011). Novel 1, 5-naphthyridine PI3Kδ inhibitors, an evaluation of WO2011075628. Expert Opin. Ther. Pat..

[7] Pierre F., Chua P.C., O’Brien S.E., Siddiqui J.A., Bourbon P., Haddach M., Michaux J., Nagasawa J., Schwaebe M.K., Stefan E. (2011). Discovery and sar of 5-(3-chlorophenylamino)benzo[c][2,6]naphthyridine-8-carboxylic acid (CX-4945), the first clinical stage inhibitor of protein kinase ck2 for the treatment of cancer. J. Med. Chem..

[8] Bilodeau M.T., Balitza A.E., Hoffman J.M., Manley P.J., Barnett S.F., Defeo J.D., Haskell K., Jones R.E., Leander K., Robinson R.G. (2008). Allosteric inhibitors of Akt1 and Akt2: A naphthyridinone with efficacy in an A2780 tumor xenograft model. Bioorg. Med. Chem. Lett..

[9] Kaila N., Green N., Li H.Q., Hu Y., Janz K., Gavrin L.K., Thomason J., Tam S., Powell D., Cuozzo J. (2007). Identification of a novel class of selective Tpl2 kinase inhibitors: 4-Alkylamino-[1, 7] naphthyridine-3-carbonitriles. Bioorg. Med. Chem..

[10] Gavrin L.K., Green N., Hu Y., Janz K., Kaila N., Li H.Q., Tam S.Y., Thomason J.R., Gopalsamy A., Ciszewski G. (2005). Inhibition of Tpl2 kinase and TNF-α production with 1, 7-naphthyridine-3-carbonitriles: Synthesis and structure–activity relationships. Bioorg. Med. Chem. Lett..

[11] Zhuo L.S., Xu H.C., Wang M.S., Zhao X.E., Ming Z.H., Zhu X.L., Huang W., Yang G.F. (2019). 2, 7-naphthyridinone-based MET kinase inhibitors: A promising novel scaffold for antitumor drug development. Eur. J. Med. Chem..

[12] Wang Y., Xu Z.L., Ai J., Peng X., Lin J.P., Ji Y.C., Geng M.Y., Long Y.Q. (2013). Investigation on the 1, 6-naphthyridine motif: Discovery and SAR study of 1 H-imidazo [4, 5-h][1, 6] naphthyridin-2 (3 H)-one-based c-Met kinase inhibitors. Organic Biomol. Chem..

[13] Wang M.S., Zhuo L.S., Yang F.P., Wang W.J., Huang W., Yang G.F. (2019). Synthesis and biological evaluation of new MET inhibitors with 1, 6-naphthyridinone scaffold. Eur. J. Med. Chem..

[14] Gibson S.A., Benveniste E.N. (2018). Protein kinase CK2: An emerging regulator of immunity. Trends Immunol..

[15] Madak J.T., Cuthbertson C.R., Miyata Y., Tamura S., Petrunak E.M., Stuckey J.A., Han Y.Y., He M., Sun D.X., Showalter H.D. (2018). Design, synthesis, and biological evaluation of 4-quinoline carboxylic acids as inhibitors of dihydroorotate dehydrogenase. J. Med. Chem..

[16] Kumar V., Jaggi M., Singh A.T., Madaan A., Sanna V., Singh P., Sharma P.K., Irchhaiya R., Burman A.C. (2009). 1, 8-Naphthyridine-3-carboxamide derivatives with anticancer and anti-inflammatory activity. Eur. J. Med. Chem..

[17] Fu L., Feng X., Wang J.J., Xun Z., Hu J.D., Zhang J.J., Zhao Y.W., Huang Z.B., Shi D.Q. (2015). Efficient synthesis and evaluation of antitumor activities of novel functionalized 1,8-naphthyridine derivatives. ACS Comb. Sci..

[18] Manera C., Malfitano A.M., Parkkari T., Lucchesi V., Carpi S., Fogli S., Bertini S., Laezza C., Ligresti A., Saccomanni G. (2015). New quinolone-and 1, 8-naphthyridine-3-carboxamides as selective CB2 receptor agonists with anticancer and immuno–modulatory activity. Eur. J. Med. Chem..

[19] Gross H., Goeger D.E., Hills P., Mooberry S.L., Ballantine D.L., Murray T.F., Valeriote F.A., Gerwick W.H. (2006). Lophocladines, Bioactive Alkaloids from the Red Alga Lophocladia sp.. J. Nat. Prod..

[20] Kumpan K., Nathubhai A., Zhang C., Wood P.J., Lloyd M.D., Thompson H.T., Lehtio L., Threadgill M.D. (2015). Structure-based design, synthesis and evaluation in vitro of arylnaphthyridinones, arylpyridopyrimidinones and their tetrahydro derivatives as inhibitors of the tankyrases. Bioorg. Med. Chem..

[21] Theeramunkong S., Vajragupta O., Mudjupa C. (2016). Synthesis and biological evaluation of simplified analogs of lophocladine B as potential antitumor agents. Med. Chem. Res..

[22] Zhang A., Ding C., Cheng C., Yao Q. (2007). Convenient synthesis of 2, 7-naphthyridine lophocladines A and B and their analogues. J. Comb. Chem..

[23] Gopalsamy A., Shi M., Boschelli D.H., Williamson R., Olland A., Hu Y., Krishnamurthy G., Han X., Arndt K., Guo B. (2007). Discovery of dibenzo[c,f][2,7]naphthyridines as potent and selective 3-phosphoinositide-dependent kinase-1 inhibitors. J. Med. Chem..

[24] Zhang Y., Illarionov B., Bacher A., Fischer M., Georg G.I., Ye Q.Z., Vander V.D., Fanwick P.E., Song Y., Cushman M. (2007). A novel lumazine synthase inhibitor derived from oxidation of 1, 3, 6, 8-tetrahydroxy-2, 7-naphthyridine to a tetraazaperylenehexaone derivative. J. Org. Chem..

[25] Ukita T., Nakamura Y., Kubo A., Yamamoto Y., Moritani Y., Saruta K., Higashijima T., Kotera J., Fujishige K., Takagi M. (2003). 1, 7-and 2, 7-naphthyridine derivatives as potent and highly specific PDE5 inhibitors. Bioorg. Med. Chem. Lett..

[26] Kim H.O., Ryu J.H., Jung J.Y., Lee N.K., Kim J.H., Kim E.J., Joen S.D., Kim J.H., Rhee H.I., Cho Y.B. (2006). Pyridine Derivatives, Methods of their Preparations, and Pharmaceutical Compositions Containing the Same. PTC Int. Appl. WO.

[27] Okram B., Uno T., Ding Q., Liu Y.H., Jin Y.H., Jin Q.H., Wu X., Chen J.W., Yan S.F. (2009). Compounds and Compositions as Kinase Inhibitors. PTC Int. Appl. WO.

[28] Ziegler D.S., Greiner R., Lumpe H., Kqiku L., Karaghiosoff K., Knochel P. (2017). Directed zincation or magnesiation of the 2-pyridone and 2, 7-naphthyridone scaffold using TMP bases. Org. Lett..

[29] Kumar R., Knick V.B., Rudolph S.K. (2007). Pharmacokinetic-pharmacodynamic correlation from mouse to human with pazopanib, a multikinase angiogenesis inhibitor with potent antitumor and antiangiogenic activity. Mol. Cancer Ther..

[30] Cui J.J. (2014). Targeting receptor tyrosine kinase MET in cancer: Small molecule inhibitors and clinical progress. J. Med. Chem..

[31] Morris G.M., Huey R., Lindstrom W., Sanner M.F., Belew R.K., Goodsell D.S., Olson A.J. (2009). AutoDock4 and AutoDockTools4: Automated docking with selective receptor flexibility. J. Comput. Chem..

[32] Gasteiger J., Marsili M. (1980). Iterative partial equalization of orbital electronegativity—A rapid access to atomic charges. Tetrahedron.

[33] Morris G.M., Goodsell D.S., Halliday R.S., Huey R., Hart W.E., Belew R.K., Olson A.J. (1998). Automated docking using a Lamarckian genetic algorithm and an empirical binding free energy function. J. Comput. Chem..

[34] Berman H.M., Westbrook J., Feng Z., Gilliland G., Bhat T.N., Weissig H., Shindyalov I.N., Bourne P.E. (2000). The protein data bank. Nucleic Acids Res..

[35] Garner A.P., Gozgit J.M., Anjum R., Vodala S., Schrock A., Zhou T., Serrano C., Eilers G., Zhu M., Ketzer J. (2014). Ponatinib inhibits polyclonal drug-resistant KIT oncoproteins and shows therapeutic potential in heavily pretreated gastrointestinal stromal tumor (GIST) patients. Clin. Cancer Res..

[36] Tasker A.S., Patel V.F. PDB bank [3EFL].

[37] The PyMOL Molecular Graphics System, Version 1.2r3pre, Schrödinger, LLC. https://pymol.org/2/.

